# Deficiency of Nuclear Receptor Nur77 Aggravates Mouse Experimental Colitis by Increased NFκB Activity in Macrophages

**DOI:** 10.1371/journal.pone.0133598

**Published:** 2015-08-04

**Authors:** Anouk A. J. Hamers, Laura van Dam, José M. Teixeira Duarte, Mariska Vos, Goran Marinković, Claudia M. van Tiel, Sybren L. Meijer, Anne-Marieke van Stalborch, Stephan Huveneers, Anje A. te Velde, Wouter J. de Jonge, Carlie J. M. de Vries

**Affiliations:** 1 Department of Medical Biochemistry, Academic Medical Center, University of Amsterdam, Amsterdam, The Netherlands; 2 Tytgat Institute, Academic Medical Center, University of Amsterdam, Amsterdam, The Netherlands; 3 Department of Pathology, Academic Medical Center, University of Amsterdam, Amsterdam, The Netherlands; 4 Department of Molecular Cell Biology, Sanquin Research and Swammerdam Institute for Life Sciences, University of Amsterdam, Amsterdam, The Netherlands; IRCCS Istituto Oncologico Giovanni Paolo II, ITALY

## Abstract

Nuclear receptor Nur77, also referred to as NR4A1 or TR3, plays an important role in innate and adaptive immunity. Nur77 is crucial in regulating the T helper 1/regulatory T-cell balance, is expressed in macrophages and drives M2 macrophage polarization. In this study we aimed to define the function of Nur77 in inflammatory bowel disease. In wild-type and Nur77^-/-^ mice, colitis development was studied in dextran sodium sulphate (DSS)- and 2,4,6-trinitrobenzene sulfonic acid (TNBS)-induced models. To understand the underlying mechanism, Nur77 was overexpressed in macrophages and gut epithelial cells. Nur77 protein is expressed in colon tissues from Crohn’s disease and Ulcerative colitis patients and colons from colitic mice in inflammatory cells and epithelium. In both mouse colitis models inflammation was increased in Nur77^-/-^ mice. A higher neutrophil influx and enhanced IL-6, MCP-1 and KC production was observed in Nur77-deficient colons after DSS-treatment. TNBS-induced influx of T-cells and inflammatory monocytes into the colon was higher in Nur77^-/-^ mice, along with increased expression of MCP-1, TNFα and IL-6, and decreased Foxp3 RNA expression, compared to wild-type mice. Overexpression of Nur77 in lipopolysaccharide activated RAW macrophages resulted in up-regulated IL-10 and downregulated TNFα, MIF-1 and MCP-1 mRNA expression through NFκB repression. Nur77 also strongly decreased expression of MCP-1, CXCL1, IL-8, MIP-1α and TNFα in gut epithelial Caco-2 cells. Nur77 overexpression suppresses the inflammatory status of both macrophages and gut epithelial cells and together with the *in vivo* mouse data this supports that Nur77 has a protective function in experimental colitis. These findings may have implications for development of novel targeted treatment strategies regarding inflammatory bowel disease and other inflammatory diseases.

## Introduction

Inflammatory bowel disease (IBD) represents a group of idiopathic chronic inflammatory intestinal conditions of which the two main diseases are Crohn’s disease (CD) and ulcerative colitis (UC). The highest incidences of CD and UC have been reported in northern Europe, especially the United Kingdom and Scandinavia [1,2]; and North America [2–4]. It is thought that IBD results from an excessive immune response towards the normal gut microflora in genetically susceptible individuals exposed to environmental risk factors. Therefore defects in innate immunity are at the center of both UC and CD [5]. The intestinal epithelium acts as a protective physical barrier and is actively involved in immune cell regulation. Intercellular junctions connect the intestinal epithelial cells and defects in this structure have been reported in IBD patients to lead to increased permeability [6,7]. Intestinal epithelial cells are able to take up antigen, deliver it across the cell and efficiently present it to, preferentially, gut dendritic cells [8]. In addition, these cells can express a range of inflammatory cytokines and chemokines such as tumor necrosis factor α (TNFα) and interleukin (IL)-8 via the activation of NFκB, further emphasizing their role in immune regulation [9–13]. Key immune cells that maintain intestinal homeostasis are macrophages, dendritic cells and T-cells. Dysregulation of the activation of macrophages and dendritic cells leads to development of IBD through activation of colitogenic T-cell populations. CD has been associated with an exaggerated Th1/Th17 response, while in the healthy colon these T-cell subtypes are homeostatically restrained by Foxp3^+^ regulatory T-cells (Tregs) [14,15].

Within the last era, it has become clear that next to immunological and environmental factors (altered luminal bacteria) genetic factors play an important role in the pathogenesis of IBD [16].

Nuclear receptor Nur77 is also known as NR4A1, TR3 or NGFI-B and is a member of the NR4A receptor subfamily that additionally comprises Nurr-1 (NR4A2, NOT) and NOR-1 (NR4A3, MINOR). Like other nuclear receptors, the NR4A receptors consist of an N-terminal transactivation domain, a central DNA binding domain and a C-terminal ligand binding domain. So far, no ligands have been identified for the NR4A receptors, and therefore they are referred to as orphan nuclear receptors [17]. Induction of Nur77 can be achieved upon stimulation with inflammatory factors, such as prostaglandins, TNFα, lipopolysaccharide (LPS), Interferon gamma (IFNγ) and granulocyte-macrophage colony stimulating factor (GM-CSF) [18–20].

An immunological role for Nur77 has first been demonstrated by the group of Winoto [21], showing that Nur77 is necessary for induced apoptosis in T-cells and thus appears to be functionally involved in thymocyte selection [22]. Recently, ectopic expression of Nur77 has been found to induce forkhead box P3 (Foxp3) expression and suppress effector cytokine expression in T-cell receptor stimulated CD4^+^ T-cells, proving Nur77 crucial in regulating the Th1/Treg balance [23]. Given that Nur77 also influences innate immunity, Nur77 potentially plays a role in IBD. In human THP-1 macrophages Nur77 gain of function and knockdown experiments revealed that this nuclear receptor reduces the expression of several inflammatory cytokines in response to either LPS or TNFα [18]. It has been proposed that Nur77 modulates inflammatory gene expression at least in part through transrepression of NFκB, in line with studies showing that Nur77 inhibits NFκB activity by binding its p65 subunit and enhancing IκBα expression [24–26]. Increased NFκB activation has been proven to be a causative factor in development of experimental colitis and IBD (predominantly UC and CD) [11,27,28]. Moreover, intravenously or rectally administered NFκB p65 antisense oligonucleotides ameliorate 2,4,6-trinitrobenzene sulfonic acid (TNBS)-induced colitis and colitis in IL10^-/-^ mice [28]. In line with our previous findings from macrophage cell lines, Nur77^-/-^ bone marrow derived macrophages [29] and peritoneal macrophages [30] display a pro-inflammatory M1 phenotype after LPS stimulation, which may diminish mucosal healing thereby increasing local damage in the gut. Furthermore, thioglycollate-induced migration of circulating cells to the peritoneal cavity was markedly increased in Nur77^-/-^ mice [29] and Nur77^-/-^ mice lack the Ly6C^-^ monocyte population in bone marrow, spleen and blood [31]. These monocytes patrol the resting vasculature and participate in the resolution of inflammation [32].

In endothelial cells it is shown that Nur77 reduces the expression of adhesion molecules VCAM-1 and ICAM-1, resulting in decreased monocyte adhesion [26]. In an *E*.*coli*-induced peritonitis mouse model Nur77 was shown to modulate bacterial influx into liver and lung via increased vascular permeability [33].

Given the involvement of Nur77 in innate and adaptive immunity and the fact that the function of Nur77 in the development of colitis has not yet been studied, we employed the dextran sodium sulphate (DSS)- and TNBS-induced models of colitis in WT and Nur77^-/-^ mice to assess Nur77 function in IBD. We show that Nur77^-/-^ mice have increased colon inflammation with more neutrophils, T-cells and macrophages in the lesions. Nur77 overexpression dampens the pro-inflammatory state of RAW macrophages and epithelial Caco-2 cells.

## Materials and Methods

### Mice

*Nur77* ^*-/-*^ mice [34] on a C57BL/6 background were kindly provided by prof BR Binder (Vienna, Austria). Wild-type (WT) mice with a C57BL/6 background were obtained from Jackson Laboratories. All animals were specific pathogen free. All animal experiments were approved by the Committee for Animal Welfare of Amsterdam Medical Center (Permit number 11800) and were carried out in compliance with guidelines issued by the Dutch government. Rectal infusion of 2,4,6-trinitrobenzene sulphonic acid (TNBS) was performed under isoflurane anesthesia and all efforts were made to minimize suffering. More detailed information on experimental procedures, animals and housing is available in the supporting information (S1 text).

### Nur77 protein expression in human and mouse colonic tissue

Human patient tissue paraffin sections were obtained from the IBD tissue bank at the Tytgat Institute (Academic Medical Center, Amsterdam, The Netherlands) which was obtained with approval by the Medical Ethical Committee of the Academic Medical Center in Amsterdam (METC AMC) as previously described [35,36]. Human and mouse Nur77 protein was detected with the same antibody (M210 Rabbit polyclonal; Santa Cruz Biotechnology, Santa Cruz, CA). First, antigen retrieval was performed at pH 9.0 and the sections were incubated in 1% (v/v) hydrogen peroxide (Merck, Darmstadt, Germany) followed by a blocking step. Sections were incubated with the first antibody overnight at 4°C followed by a horseradish peroxidase (HRP)-conjugated secondary Goat anti-rabbit antibody (Immunologic, Duiven, The Netherlands) for 1 hr at room temperature. 3,3'-Diaminobenzidine (DAB) substrate (ImmunoLogic) was used for detection and sections were counterstained with Mayer’s hematoxillin.

### Flow cytometry of colon macrophages

Lamina propria cell isolation from mouse colon was performed as previously described [37]. In short; colons were removed, soaked in PBS and cut in small 0.5mm segments. Tissue segments were incubated at 37°C in Hank’s balanced saline solution (HBSS; Gibco) containing 2mM ethylenediaminetetraacetic acid (EDTA; Sigma-Aldrich, St. Louis, MO) for 2 x 15 minutes. After each incubation step, vigorous shaking and discarding supernatant removed epithelial cells. Next, tissue was digested using 0.425mg/ml collagenase V (Sigma-Aldrich), 0.625mg/ml collagenase D (Roche, Mannheim, Germany), dispase 1mg/ml (GIBCO Invitrogen, Carlsbad, CA), and 30μg/ml DNase (Roche) in RPMI (GIBCO Invitrogen) supplemented with 10% heat-inactivated fetal calf serum (FCS; GIBCO Invitrogen), Penicillin/Streptomycin, L-glutamine (Sigma-Aldrich), fungizone, and 2-β-mercaptoethanol (Sigma-Aldrich) for approximately 45 min at 37°C in a shaker. During incubation, tubes were shaken vigorously every 5–7 min. Thereafter, single cell suspensions were passed through a 40μM cell strainer. After washing with PBS cells were labeled for flow cytometry with the following antibodies: CD45-APC-Cy7 (eBioscience, San Diego, CA), CD11b-PERCP (eBioscience), Ly6G-FITC (Macs), CD64-PE (eBioscience), Ly6C-APC (eBioscience) and MHCII-PE-Cy7 (eBioscience). Dead cells were excluded by DAPI. Samples were analyzed using a LSR Fortessa (BD Biosciences, San Jose, CA) and FlowJo software (Tree Star, Ashland, OR).

### DSS colitis

Colitis was induced by dextran sodium sulphate (DSS; Sigma-Aldrich). Twelve week old female WT mice and Nur77^-/-^ mice (10 per group) were exposed to 1.5% of DSS in their drinking water every day for 7 days. The mice were weighed every morning. Mice were sacrificed at day 8.

### TNBS colitis

Colitis was induced using TNBS (Sigma-Aldrich) as described previously [38,39]. Twelve week old female WT mice and Nur77^-/-^ mice (10 per group) received 1.5mg of TNBS dissolved in 40% of ethanol (Merck) intra-rectally using a vinyl catheter positioned 3cm from the anus at day 1 and day 7 to establish a Delayed-Type Hypersensitivity reaction involving innate immunity and specific T-cell response towards haptenized proteins [39]. After instillation mice were kept in a vertical position for 60 seconds. The mice were weighed every morning and sacrificed at day 10.

### Disease assessment and histology

After sacrifice, blood, colon and spleen were harvested. Stool consistency and rectal bleeding were scored based on an arbitrary scale by two researchers independently as described previously [40]. In addition to weight-loss, the disease activity index (DAI score) was calculated for each mouse (Table 1). DAI = [body weight loss + stool consistency score + rectal bleeding score] / 3 [41]. After removing the fecal material, the colon was cut open longitudinally and the wet weight was measured. Additionally, spleen weight was determined. Finally, the colon was divided longitudinally and rolled up, one half was snap frozen in liquid nitrogen and the other half was fixed in 4% formalfix. Tissue was embedded in paraffin and cut into sections of 7μm. Colon pathology was visualized with hematoxylin-eosin (H&E) stain and scored blindly by a pathologist according to a scoring system as shown in Table 2.

**Table 1:** Scoring system Disease Activity Index (DAI).

Score A		Score B		Score C	
0	<1%	0	Normal	0	Negative
1	1–5%	1	Loose droppings	2	Positive
2	6–10%	2	Loose stools, colon filled with feces	4	Gross bleeding
3	11–15%	3	Loose stools, feces only near cecum		
4	>15%	4	Empty bowel		

**Table 2:** Scoring system colon pathology.

score	0	1	2	3
Area involved	0%	1–10%	10–50%	>50%
Follicles	Normal (0–1)	Little (2–3)	Moderate (4–5)	Extensive (>6)
Edema	Absent	Little	Moderate	Extensive
Fibrosis	Absent	Little	Moderate	Extensive
Erosion/ulceration	Absent	Lamina propria	Submucosa	Transmural
Crypt loss	0%	1–10%	10–50%	>50%
Granulocytes	Normal	Few	Moderate	Extensive
Mononuclear cells	Normal	Few	Moderate	Extensive

### Immunohistochemistry

Paraffin sections were deparaffinized and rehydrated. To detect macrophages (F4/80 Rat monoclonal; clone CI A3-1; Serotec, Kidlington, United Kingdom) the sections were incubated in 1% (v/v) hydrogen peroxide (Merck) followed by antigen retrieval at pH6.0. The sections were blocked with 50% Ultra-V-Block (Immunologic) in TBS and were subsequently incubated with first antibody (1:500) overnight at 4°C followed by a horseradish peroxidase (HRP)-conjugated secondary rabbit anti-rat antibody (Immunologic). DAB substrate (ImmunoLogic) was used for detection.

T-cells were detected with an antibody against CD3 (Rabbit monoclonal; clone SP7; Lab Vision, Fremont, CA), antigen retrieval was performed at pH9.0 and the sections were blocked using 1% (v/v) normal goat serum and 1% (w/v) BSA in TBS. The first antibody was incubated at a 1:500 dilution for 1 hour at room temperature followed by an HRP-conjugated secondary Envision anti-rabbit antibody (DAKO, Glostrup, Denmark). DAB substrate was used for detection.

For neutrophils (Ly-6B.2 Rat monoclonal; clone 7/4; Serotec), no antigen retrieval was necessary. The sections were blocked using 1% (v/v) normal goat serum and 1% (w/v) BSA in TBS. The first antibody was incubated at a 1:2000 dilution for 1 hour at room temperature followed by an HRP-conjugated secondary donkey anti-rat antibody (Jackson labs, West Grove, PA). DAB substrate was used for detection.

To study cell proliferation in the gut, sections were stained for Ki67 (Rabbit polyclonal; clone SP6; Thermo Scientific, Waltham, MA). Antigen retrieval at pH 6.0 was performed before incubation in 1% hydrogen peroxide. The sections were blocked with 50% Ultra-V-Block (Immunologic) in TBS and were subsequently incubated with first antibody 1:1000 overnight at 4°C followed by a horseradish peroxidase (HRP)-conjugated secondary goat anti-rabbit antibody (Immunologic). DAB substrate was used for detection.

After counterstaining with hematoxylin the sections were embedded in pertex (HistoLab, Gothenburg, Sweden). Macrophage area was quantified using Leica QWin V3 software (Cambridge, United Kingdom). For quantification of T-cells or neutrophils the number of cells was counted manually in 5 areas/section (1 section/mouse) and a cell to surface ratio was calculated. Surface area was quantified using Leica QWin V3 software. The number of proliferating cells was counted in 10 crypts/section (1 section/mouse).

### mRNA isolation and qPCR for colon tissue

After crushing the colon under liquid nitrogen, total RNA was extracted using Trizol and cDNA was made from 1μg RNA with iScript cDNA Synthesis kit (BioRad, Hercules, CA). Semi-quantitative real-time PCR was performed using iQ SYBR Green Supermix (BioRad) and measured with the MyIQ system. Specific primers for Foxp3 and ribosomal protein 36B4 (to correct for cDNA content) were designed; see S1 Table.

## Mouse Blood Parameters

Protein levels of IL-6, IL-10, IL-12, IFNγ, MCP-1 and TNFα were measured in plasma using the Cytometric Bead Array Mouse Inflammation Kit (BD Biosciences) according to the manufacturer’s instructions.

### Cytokine measurements in colon lysate and colon culture supernatant

Colon lysates for cytokine measurements were prepared in Greenburger Lysis buffer (75mM NaCl, 7.5mM Tris, 0.5mM MgCl.H_2_O, 0.5mM CaCl_2_, 0.5% Triton (Sigma-Aldrich)) supplemented with complete miniprotease inhibitor cocktail (Roche). A DC protein assay was performed in order to correct for total protein. A 5mm piece of proximal colon was washed in RPMI containing 100U/ml penicillin/streptomycin, placed in 500μl RPMI supplemented with 10% FCS and penicillin/streptomycin, and incubated for 24 hours at 37°C 5% CO2. Supernatant was collected and the colon pieces were weighed.

Protein levels of IL-6, IL-10, IL-12, IFNγ, MCP-1 and TNFα were measured in colon tissue lysate and colon culture supernatant using the Cytometric Bead Array Mouse Inflammation Kit (BD Biosciences) according to the manufacturer’s instructions. Chemokine ligand 1 (KC/CXCL1) levels in culture supernatants were detected by ELISA using Duo-set antibodies according to the manufacturer’s instructions (R&D Systems, Abington, United Kingdom).

### Cell culture, transfection and qPCR

RAW264.7 mouse macrophages (ATCC) were cultured in RPMI containing 100U/ml penicillin/streptomycin (GIBCO Invitrogen) and 10% FCS. Cells were transfected either with a control plasmid (pCMV-myc) or pCMV-myc-Nur77 using the LTX lipofectamine kit (Invitrogen) according to the manufacturer’s instructions for this specific cell line. After 24 hours cells were stimulated with 50ng/ml LPS.

Caco-2 human gut epithelial cells (p41–55; ATCC) were cultured in DMEM-HG (GIBCO Invitrogen) supplemented with 100U/ml penicillin/streptomycin, 20% FCS (GIBCO Invitrogen) and 4mM L-glutamine (Sigma-Aldrich) and were passaged when 50% confluent. [42] Cells were transfected either together with pCMV-myc or pCMV-myc-Nur77 [33] using the LTX lipofectamine kit (Invitrogen) according to the manufacturer’s instructions for this specific cell line. Cells were stimulated with 25ng/ml IL-1β.

RNA was extracted with Aurum Total RNA Mini Kit (BioRad), cDNA was synthesized from 500ng total RNA with iScript cDNA Synthesis kit (BioRad), and semi-quantitative real-time PCR was performed using iQ SYBR Green Supermix (BioRad) and was measured with the MyIQ system. Specific primers for mouse IL-10, KC, IL-6, KC, CX3CR1, MCP-1, TNFα, MIF and ribosomal protein 36B4 (to correct for cDNA content) or human Nur77, MCP-1, MIP-1α, TNFα, CXCL1, Claudin-2 (Cldn-2), Occludin (Ocln), IL-6, IL-8 and ribosomal protein 36B4 (to correct for cDNA content) were designed (for primer sequences see S1 Table).

### NFκB transcriptional activity

RAW264.7 stable cell line containing the 3x-NFκBluc plasmid [43] was kindly provided by Prof Menno de Winther [44]. Cells were cultured in DMEM-high glucose containing 100U/ml penicillin/streptomycin (GIBCO Invitrogen) and 10% FCS. Cells were transfected with pCMV-myc or pCMV-myc-Nur77 as described above. pRL-TK Renilla reporter plasmid (Promega) was co-transfected as an internal control for transfection efficiency. After 24 hours cells were stimulated with 100ng/ml LPS for 4 hours and luciferase activity was determined using the dual-luciferase reporter assay system (Promega) according to the manufacturer’s instructions.

Caco-2 cells were transfected with two different NFκB luciferase reporter plasmids (pLuc-NFκB-Stratagene (La Jolla, CA) and pKB3-IL6-luc) and either pCMV-myc or pCMV-myc-Nur77 using the LTX lipofectamine kit (Invitrogen) according to the manufacturer’s instructions for this specific cell line. The construct containing the NFκB response element of the minimal IL-6 promoter (pKB3-IL6-luc) was kindly provided by Dr. Karolien De Bosscher, Ghent University, Belgium [45]. hNur77 cDNA (GenBank D49728, bp 8–1947) was amino-terminally epitope tagged by cloning into the pCMV-myc vector (Clontech, Mountain View, CA). pRL-TK renilla reporter plasmid (Promega, Madison WI) was co-transfected as an internal control. 24 Hours after transfection cells were stimulated with 25ng/ml IL-1β and luciferase activity was determined 24h later using the dual-luciferase reporter assay system (Promega) according to the manufacturer’s protocol

### Immunoblotting

Caco-2 were transfected and 24 hours later the cells were washed twice with PBS and lysed in ice-cold NP-40 lysis buffer (50nM Tris-HCl ph7.4, 100mM NaCl, 10mM NaF, 1mM Na_3_PO_4_, 10% glycerol, 1% nonidet). After a 10 minute incubation on ice the lysates were collected, sonicated for 1 minute and boiled in sample buffer containing DTT. Samples were thereafter analysed by SDS-PAGE. Zona occludens protein 1 (ZO-1) was separated on 7.5% polyacrylamide SDS gels and the other proteins on 12% gels. Proteins were transferred to 0.2μm nitrocellulose membranes (Whatman) using the Trans-blot Turbo transfer system (Biorad). ZO-1 was transferred by overnight wet blotting at 4°C. Blots were subsequently blocked in 5% (w/v) non-fat milk in Tris-buffered saline (TBS) and incubated with specific primary antibodies overnight at 4°C, followed by a horse radish peroxidase-labelled secondary antibody (Biorad) for 1 hour at room temperature. The following primary antibodies were used: anti-ZO-1 (Invitrogen), anti-occludin (Invitrogen), anti-β-catenin (Cell Signalling, Leiden, The Netherlands), anti-claudin-5 (Zymed), anti-E-cadherin (Cell Signalling), and β-actin (Cell Signalling). Proteins were visualized with an enhanced chemiluminescence (ECL) detection system (Thermoscientific) and quantification of signals was performed using intensity measurements in ImageJ software.

### Permeability assay

Monolayer formation and barrier function were determined by measuring the electrical trans-epithelial resistance (TER) with electric cell-substrate impedance sensing (ECIS). 1.25*10^5^ Transfected Caco-2 cells were seeded on the gold electrode arrays (8W10E; IBIDI, Planegg, Germany) treated with 10mM L-cysteine (Sigma) for 15 minutes at 37°C and coated with fibronectin. Measurements were started directly after seeding the cells and impedance measurements were performed at 4kHz every 5 minutes.

### Statistical analysis

Statistical analysis was performed using GraphPad Prism 5 software (La Jolla, CA). Statistical significance was calculated using the unpaired Student’s T-test (Welch corrected when necessary). Values are represented as mean ± SEM or SD as indicated in the figure legends. The significance level was set at p<0.05.

## Results

### Nur77 protein is expressed in colon of patients with Crohn’s disease (CD) or ulcerative colitis (UC) and in diseased mouse colon

**Fig 1:**
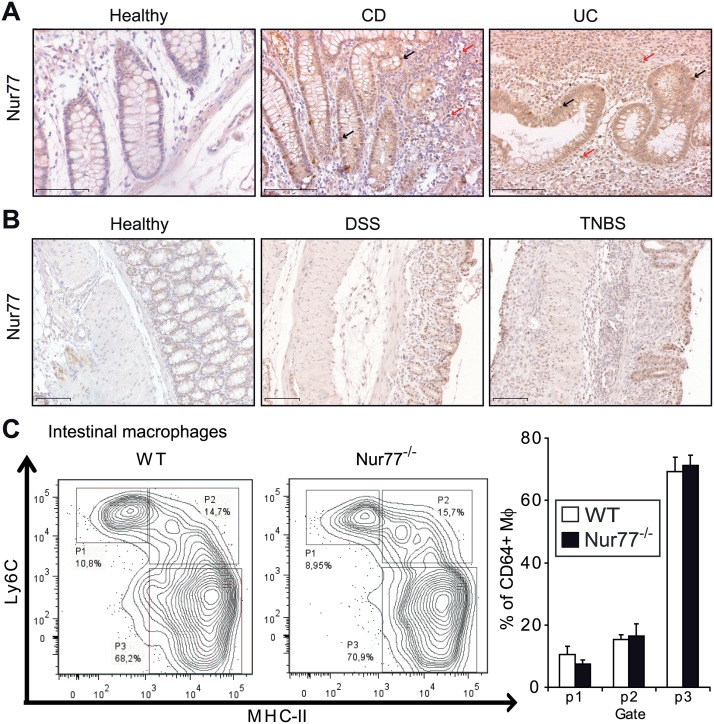
Nur77 is expressed in inflamed human and mouse colon tissue and intestinal macrophage populations are similar in healthy colons of WT and Nur77^-/-^ mice. Immunohistochemistry for Nur77 was performed on colon sections from a healthy and affected part of the colon from patients (A) with either Crohn’s disease (CD) or ulcerative colitis (UC) and colon sections from WT mice (B) before and after DSS- or TNBS-induced colitis. Representative photomicrographs are shown with original magnification 200x (A), 100x (B). Arrows indicate examples of cells with nuclei positive for Nur77 protein in epithelial cells (black) and inflammatory cells (red). Colonic lamina propria cells were isolated for flow cytometry and macrophages were selected and presented as three different subpopulations (C). Data are presented as mean±SD. Scale bars represent 1mm.

In previous studies we have demonstrated expression of Nur77 in human atherosclerosis, which may be considered a chronic inflammatory disease of the vessel wall [18,46]. In the current study we show for the first time Nur77 protein expression in colon tissue from CD and UC patients (Fig 1A). In healthy colon epithelium Nur77 expression was very low compared to diseased tissue. The nuclei of infiltrated inflammatory cells were clearly positive for Nur77 in the CD and UC tissue sections. In colon from TNBS- and DSS-treated mice Nur77 was expressed in inflammatory cells, very prominently in the epithelium, and in smooth muscle cells of the muscularis externa (Fig 1B).

### Nur77-deficiency does not influence macrophage populations in the colon

In the gut, resident intestinal macrophages do not express the lipopolysaccharide (LPS) co-receptor CD14 or the IgA (CD89) and IgG (CD16, 32, and 64) receptors, yet TLRs 3–9 are present. These cells can be distinguished from inflammatory monocytes on the basis of high CX3CR1 and CD64 expression. Nur77^-/-^ mice lack the CX3CR1-expressing Ly6C^-^ monocyte population in bone marrow, circulation and spleen [31]. Therefore, we set out to investigate the different macrophage populations present in colon from WT and Nur77^-/-^ mice by flow cytometry. The gating strategy was set-up according to the phenotypic characterization of colonic macrophages by Mowat and co-workers (S1 Fig) [37]. Within the live CD45^+^ population of colonic lamina propria cells, the CD11b^+^CD64^+^Ly6G^-^ cells were selected and further characterized. Three different macrophage populations, each with a different pattern of Ly6C and MHC-II expression, were observed (Fig 1C). Population 1 (p1; Ly6C^hi^ MHC-II^-^), p2 (Ly6C^hi^ MHC-II^+^) and p3 (Ly6C^lo^ MHC-II^+^) were all similar in WT and Nur77^-/-^ mice (Fig 1C). Based on these analyses, we propose that even though Nur77^-/-^ mice have a monocyte subset-phenotype the gut macrophage subsets appeared to be normal and similar in WT and Nur77^-/-^ mice in a healthy setting.

### Nur77^-/-^ mice show more inflammation in DSS-induced colitis

**Fig 2:**
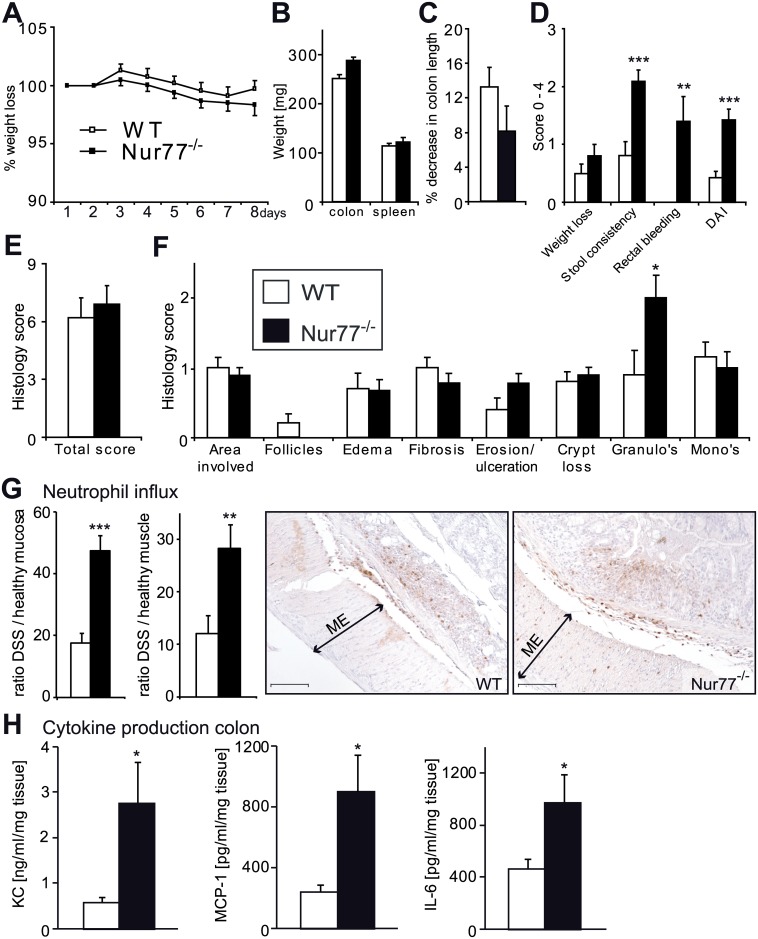
Nur77^-/-^ mice develop augmented DSS-induced colitis. The body weight of the mice was determined daily during DSS treatment (A). Upon harvest colon weight, spleen weight (B) and colon length (C) were determined. The disease activity index (DAI) was calculated as an average in weight loss, stool consistency and bleeding scores (D). Colon histology was scored on hematoxillin-eosin stained sections (E) for the parameters shown (F). Neutrophil numbers were counted in healthy and DSS colon sections and the influx was calculated by taking the ratio of Healthy / DSS (G). Representative photomicrographs are shown with original magnification 100x. Colon pieces were cultured overnight and cytokine levels were determined in the supernatant and corrected for mg of tissue (H). Data are presented as mean±SEM; *p<0.05, **p<0.01, ***p<0.001. Scale bars represent 1mm. ME = Muscularis externa, KC = Chemokine ligand 1 (mouse homologue of IL-8; a potent neutrophil attractant).

To assess whether Nur77 is functionally involved in colitis, experimental colitis was induced by DSS, establishing an acute and mild form of colitis characterized by ulceration and submucosal inflammation resembling UC in humans [47]. We choose a mild DSS 1.5% treatment because of the possibility that Nur77 deletion augments the already existing colitis. Over time, the Nur77^-/-^ and WT mice both showed only a moderate weight loss after the DSS challenge (Fig 2A). No difference was found in colon weight or spleen weight between the groups (Fig 2B). As a result of DSS-treatment, colon length was decreased, which was not different between WT and Nur77^-/-^ mice (Fig 2C). However, the scores of stool consistency and rectal bleeding (Table 1) were significantly higher in Nur77^-/-^ mice (Fig 2D). Taking the weight loss, stool consistency and rectal bleeding scores together to calculate the DAI, we concluded that overall disease is worsened in Nur77^-/-^ mice compared with WT mice. Moreover, a histological score performed on H&E colon sections showed that although the overall histology scores did not differ between WT and Nur77^-/-^ mice there was a higher influx of granulocytes in the colon of Nur77^-/-^ mice (Fig 2E and 2F). Histological analysis of colon sections from healthy WT and Nur77^-/-^ controls revealed no signs of inflammation or mucosal damage (data not shown).

To further investigate the increased granulocyte influx an immunohistochemical staining for neutrophils was performed and quantified for healthy and DSS mice. The fold increase of neutrophils was calculated which indeed revealed a significantly higher neutrophil influx in both the mucosa and the muscularis externa of Nur77^-/-^ colons (Fig 2G). To further examine the cause of this increased migration, we investigated expression of the neutrophil attracting chemokine KC in the supernatants of colonic explant cultures from DSS-treated animals and observed markedly increased KC protein levels in Nur77^-/-^ colon supernatants (Fig 2H). SDF-1α (CXCL12), another chemokine that can attract neutrophils, was not different (S2A Fig). In addition, we determined protein levels of several cytokines known to be involved in colon inflammation and observed significantly higher levels of IL-6 and MCP-1 in the supernatant of the colon explant cultures of Nur77^-/-^ compared to WT mice (Fig 2H). Systemic inflammation was not different between WT and Nur77^-/-^ mice as shown by similar IL-12, IL-6, TNFα, MCP-1, and IFNγ cytokine levels in plasma of both mouse lines (S2B Fig). Collectively, these data show a worsened outcome for Nur77^-/-^ mice in DSS-induced colitis with increased expression of inflammatory chemokines locally in the gut that are likely contributing to the enhanced neutrophil influx, and augmented disease.

### Nur77^-/-^ mice show increased inflammation in TNBS-induced colitis associated with an increased recruitment of T-cells and inflammatory monocytes

**Fig 3:**
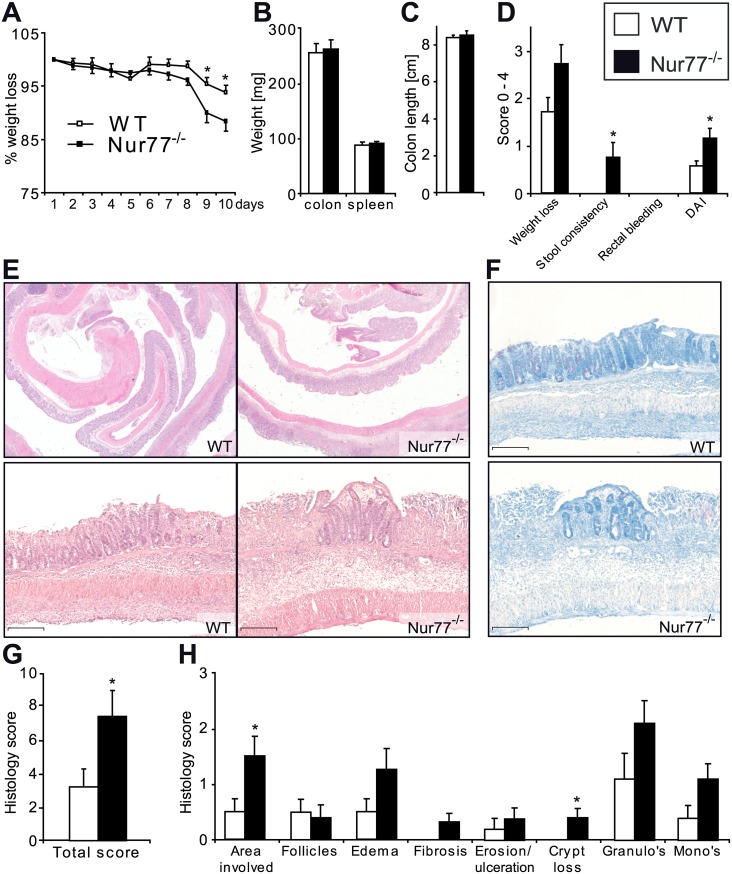
Nur77 deficiency increases TNBS-induced colitis. The body weight of the mice was determined daily from the onset of the experiment (A). Upon harvest colon weight, spleen weight (B) and colon length (C) were determined. The disease activity index (DAI) was calculated as an average in weight loss, stool consistency and bleeding scores (D). Colon histology was scored on hematoxillin-eosin stained sections (E, G) for the parameters shown (H). To illustrate the crypt loss colon sections were stained with toluidine blue (F). Representative photomicrographs are shown with original magnification 12.5x (E upper panel) and 100x (E lower panel, F). Data are presented as mean±SEM; *p<0.05. Scale bars represent 1mm.

We next investigated the role of Nur77 in TNBS-induced colitis, which develops as a delayed-type hypersensitivity reaction towards haptenized proteins, to assess the effect of Nur77 deficiency in an adaptive immune response involving T-cells and macrophages [41,48]. In this model, the mice lost more weight when compared to the DSS-experiment and significant differences were seen between WT and Nur77^-/-^ mice on day 9 and 10 (Fig 3A). Colon and spleen weight and colon length (Fig 3C and 3D) did not differ between both groups. We did however observe a significant increase in the DAI score of the Nur77^-/-^ mice (Fig 3D) mainly attributed to the higher stool consistency score. The histological analysis performed on H&E colon sections showed that the overall histology score was significantly higher for the Nur77^-/-^ mice (Fig 3E–3G). These mice showed a larger inflamed area, which is indicated as area involved, and markedly increased crypt loss (Fig 3F). A toluidine blue cytochemical staining clearly showed the difference in crypt loss between the WT and Nur77^-/-^ mice (Fig 3H).

**Fig 4:**
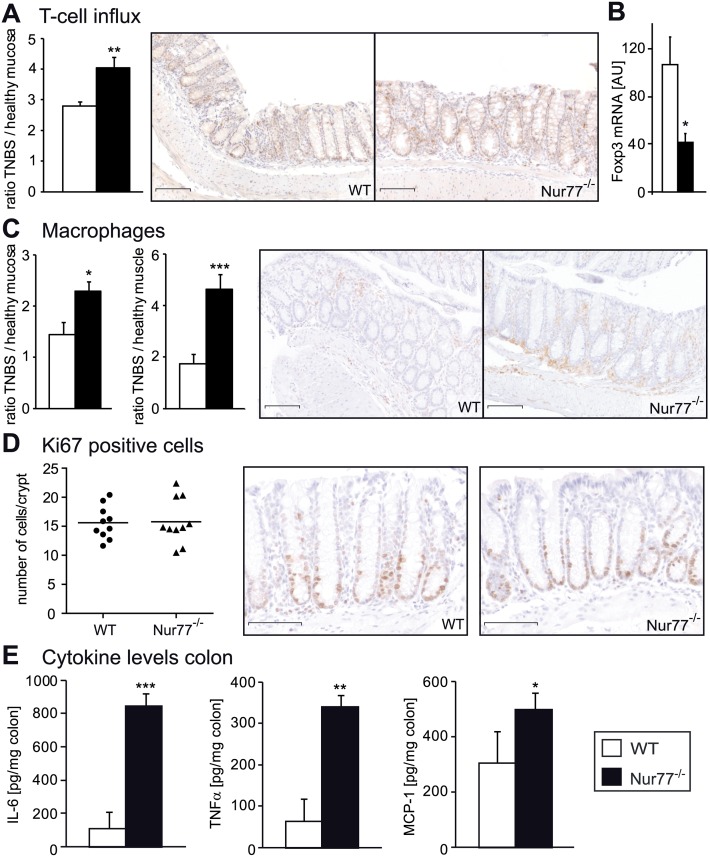
Nur77^-/-^ mice show more macrophage and T-cell influx in TNBS-induced colitis. The numbers of T-cells in the mucosa were counted and the influx was calculated by taking the ratio of Healthy / TNBS. Representative photomicrographs of sections stained for T-cells (anti-CD3) are shown (A). mRNA was extracted from colon and qPCR was performed for FoxP3 (B). cDNA levels were normalized for 36B4 housekeeping gene expression. Macrophage surface was quantified in the mucosa and the number of macrophages was counted in the muscle. The ratio of Healthy / TNBS was calculated. Representative photomicrographs of sections stained for macrophages (anti-F4/80) are shown (C). The numbers of Ki67 positive cells were counted per crypt and representative photomicrographs are shown (D). Colon lysates were prepared to determine IL-6, TNFα, and MCP-1 levels in the colon (E). A and C Original magnification x100, D x200. Each symbol represents 1 animal. Data are presented as mean±SEM; *p<0.05, **p<0.01, ***p<0.001. Scale bars represent 1mm. nr = number, AU = Arbitrary units, FoxP3 = Forkhead box P3 (marker for regulatory T-cells).

To characterize the cellular composition of the inflammation, we performed immunohistochemical stainings for T-cells and macrophages on colon sections from WT and Nur77^-/-^ mice (Fig 4A and 4C). Again, the fold increase in cell numbers relative to healthy colons was calculated. The influx of T-cells in the mucosa was significantly higher in Nur77^-/-^ mice (Fig 4A), whereas in the muscularis externa the T-cell numbers were similar (data not shown). The mRNA expression of the regulatory T-cell marker Foxp3 was significantly lower in colons of Nur77^-/-^ mice (Fig 4B). In both the mucosa and muscularis externa we found a significantly higher increase of macrophages in the Nur77^-/-^ mice than in the WT mice (Fig 4C). Regarding recent data indicating increased crypt epithelial cell proliferation in Nur77^-/-^ mice resulting in cancer development [49], we investigated the number of proliferating cells by Ki67 immunohistochemical staining in the non-inflamed crypts and no difference was found between WT and Nur77^-/-^ mice (Fig 4D).

In protein lysates from the diseased colons, the expression levels of IL-6, TNFα and MCP-1 were markedly increased in Nur77^-/-^ colons (Fig 4E). Systemic inflammation was not different between WT and Nur77^-/-^ mice as shown by similar IL-12, IL-6, TNFα, MCP-1 and IFNγ cytokine levels in plasma of both groups (S2C Fig). In conclusion, Nur77^-/-^ mice show increased inflammation of the colon after TNBS-induced colitis with more macrophages and T-cells.

### Nur77 reduces the pro-inflammatory phenotype of macrophages and inhibits the inflammatory response of the gut epithelial cell line Caco-2

**Fig 5:**
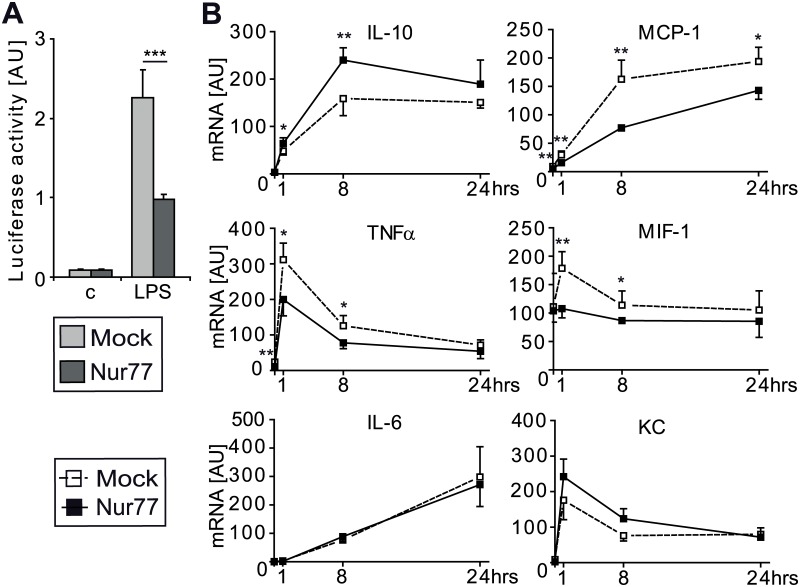
Inflammatory response of RAW264.7 macrophages after stimulation and overexpression of Nur77. RAW 3xNFκB stable cells (A) and RAW cells (B) were transfected with a plasmid encoding Nur77 or an empty plasmid (Mock) and stimulated with 100ng/ml LPS for 4 hrs or with 50ng/ml LPS for the indicated time points, respectively. NFκB activity (A) and mRNA expression of the anti-inflammatory cytokine IL-10, pro-inflammatory cytokines (TNFα and IL-6) and chemokines (MCP-1, MIF-1, KC) was determined (B). cDNA levels were normalized using 36B4 housekeeping gene. Data are presented as mean±SD; *p<0.05, **p<0.01, ***p<0.001.

Since Nur77 is known to influence the inflammatory status of macrophages [18,29,30,50], we determined NFκB activity and inflammatory gene expression in RAW macrophages. Overexpression of Nur77 repressed LPS-induced NFκB activity by 2-fold (Fig 5A). In addition, Nur77 overexpression in LPS-stimulated RAW macrophages increased expression of the protective cytokine IL-10 compared to control-infected cells (Fig 5B). Expression of the pro-inflammatory factors TNFα and MIF-1 was reduced, whereas IL-6 and KC expression was not influenced by Nur77 overexpression in these cells. MCP-1 expression in macrophages was markedly suppressed by Nur77 (Fig 5B), in line with the increased protein levels observed in Nur77^-/-^ diseased colon (Fig 4E).

**Fig 6:**
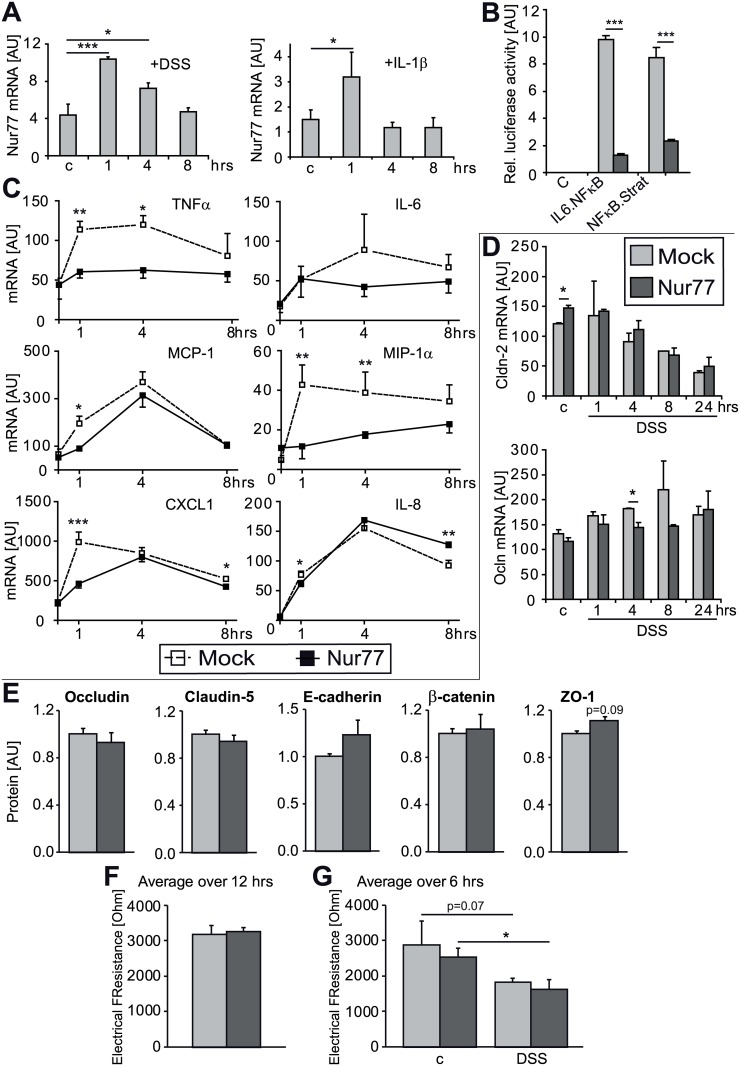
Inflammatory response of Caco-2 gut epithelial cells. Caco-2 cells were stimulated with either 1.5% DSS or 25ng/ml IL-1β and mRNA expression of endogenous Nur77 was determined (A). Caco-2 cells were transfected with two different NFκB luciferase reporter plasmids (pLuc-NFκB-Stratagene or pKB3-IL6-luc) and Nur77-encoding or empty plasmid (Mock). After 24 hours of IL-1β stimulation, luciferase activity was determined (B). Renilla reporter plasmid was co-transfected as an internal control for transfection efficiency. Caco-2 cells were transfected with Nur77 or Mock and mRNA expression of pro-inflammatory cytokines (TNFα and IL-6) and chemokines (MCP-1, MIP-1α, CXCL1, and IL-8) after IL-1β stimulation was measured (C). mRNA expression of the junction proteins claudin-2 (Cldn-2) and occludin (Ocln) was determined after 1.5% DSS treatment (D). cDNA content was normalized for 36B4 housekeeping gene expression. Levels of junction proteins occludin, claudin-5, E-cadherin, β-catenin and zona occludens protein-1 (ZO-1) were established by westernblot analysis (E). All proteins were corrected for β-actin. Barrier function was evaluated by electrical cell-substrate impedance sensing (ECIS) over a period of 12 hrs (F) after which the cells were stimulated with 1.5% DSS (G). Data are presented as mean±SD; *p<0.05, **p<0.01, ***p<0.001. AU = Arbitrary Units.

Gut epithelial cells are the first cells to respond to bacterial invasion of the colon tissue, thus we set out to investigate the role of Nur77 in Caco-2 cells. Upon treatment of Caco-2 cells, with 1.5% DSS or the NFκB activator IL-1β, Nur77 mRNA was transiently increased (Fig 6A). By measuring the luciferase activity of two different NFκB promotor constructs in response to an IL-1β stimulus in combination with Nur77 overexpression, we demonstrated that Nur77 represses NFκB activity in epithelial cells as has been shown previously in endothelial cells [26] (Fig 6B). Most NFκB-responsive genes tested showed a strong and transient induction of expression in Caco-2 cells after IL-1β stimulation with optimal expression after 1–4 hours, which diminished again after 8 hours (Fig 6C). TNFα and MCP-1 expression was repressed by Nur77 as well as the neutrophil attracting chemokines CXCL1, IL-8 and MIP-1α (Fig 6C). These data suggest that the pro-inflammatory status of gut epithelial cells in Nur77^-/-^ mice were, at least partly, responsible for the increased cellular influx observed in the colon of these mice.

### Nur77 does not affect gut epithelial cell permeability

Nur77 has been shown to influence endothelial permeability by decreasing the adherens- and tight junction proteins [33,51]. Unexpectedly, Nur77-overexpression did not affect mRNA expression of the colon epithelial junction proteins occludin and claudin-2 (Fig 6D). In addition, westernblots showed that occludin, claudin-5, E-cadherin, β-catenin did not change upon Nur77-overexpression (Fig 6E). To functionally confirm this, we have tested Caco-2 monolayer permeability by resistance measurements using ECIS, again showing no effect of Nur77 on permeability (Fig 6F). After a 1.5% DSS stimulus, the electrical resistance decreased as expected, yet no difference was seen between Mock and Nur77. Interestingly, Nur77 does enhance the permeability of endothelial cell monolayers [33].

## Discussion

In the present study we describe our findings that provide insight into the function of Nur77 in IBD. Nur77 protein is expressed in inflammatory cells and epithelium of both colon biopsies taken from IBD patients and colon sections from colitic mice. Lundin et al [52] already showed that Nur77 mRNA expression in gut epithelium is higher in conventionally raised specific pathogen-free mice compared to germ-free mice.

Recently, it has been demonstrated that Nur77 has a crucial function in differentiation of Ly6C^lo^ monocytes, which lack in Nur77^-/-^ mice [31]. Here we show that Nur77 does not seem to play a role in macrophage differentiation in the healthy colon, because flow cytometric analysis of colonic lamina propria cells revealed similar Ly6C/MHC-II subpopulations in WT and Nur77^-/-^ mice. This observation corresponds with literature suggesting that resident and pro-inflammatory macrophage subtypes in the colon all arise from Ly6C^hi^ monocytes [37,53]. Recent experiments on the role of Nur77 in muscle regeneration in mice support the theory that Nur77 is required for Ly6C^lo^ monocyte development, but that tissue Ly6C^lo^ macrophages are not generated from this monocyte subtype in response to injury [54]. Similarly as in myocardial infarction, we observed increased monocyte infiltration in Nur77^-/-^ mice in diseased colon in the TNBS-model [55].

Nur77 has been reported to exhibit a protective function in atherosclerosis, which is a disease that may be considered as a chronic inflammation of the vessel wall [29,30], whereas its role in an acute infection such as *E*.*coli*-induced peritonitis is limited [33]. We studied the effect of Nur77-deficiency in the well-established DSS- and TNBS-induced colitis mouse models and the overall observation in both models was that Nur77^-/-^ mice develop more severe disease, although with a different pathology. This is most likely due to the different ways of colitis induction; the model of TNBS colitis we have utilized develops as a delayed-type hypersensitivity reaction to haptenized proteins with T-lymphocyte mediated mucosal damage [56], whereas DSS colitis is the result of disruption of the epithelial barrier function and direct mucosal damage [57]. In the current study a relatively low dose of DSS was applied causing a milder inflammation than seen in the TNBS model, which is also reflected in higher weight loss in the latter model. DSS colitis in Nur77^-/-^ mice resulted in increased neutrophil numbers in the Nur77^-/-^ colon compared to WT colon. Neutrophils have been proven to be main contributors to gastrointestinal injury and inflammation in IBD patients and are amongst the first cells found in the gut mucosa [58–60]. These cells can even migrate from the mucosa across the gut epithelium and the number of neutrophils directly correlates with clinical disease activity and epithelial injury in patients [61,62]. Yu et al [63] found that DSS treatment massively increases the number of neutrophils in the mouse colon. The augmented neutrophil numbers we found may be attributed to increased levels of KC (mouse equivalent to IL-8) produced by the inflamed colon of Nur77^-/-^ mice compared to WT mice. As clearly shown by Alex et al [41] KC is increased in the DSS model and not after TNBS administration. Nur77 overexpression in Caco-2 cells diminished CXCL1/IL-8 production in an NFκB-dependent manner, while this was not the case for KC expression in RAW macrophages. In line with published data, the induction of CXCL1 and IL-8 mRNA in Caco-2 cells in response to a pro-inflammatory stimulus was fast and transient [64,65]. These data suggest that the gut epithelial cells are the source of the increased KC levels measured in Nur77^-/-^ DSS colons, although many other cells can produce this chemokine. Moreover, Kucharzik et al [66] demonstrated that acute induction of human IL-8 expression restricted to mouse intestinal epithelial cells is sufficient to elicit neutrophil recruitment into the lamina propria. In Caco-2 cells Nur77 also repressed MIP-1α expression, another neutrophil attracting chemokine. Unlike previously shown in endothelial cells [33,67], Nur77 did not affect expression of epithelial junction proteins in Caco-2 cells, nor did it influence monolayer permeability.

TNBS induced more colon inflammation in Nur77^-/-^ mice reflected by an increased DAI and histological score. Since this model is characterized by dense infiltration of macrophages and T-cells throughout the colon wall [38], we determined both macrophage and T-cell numbers in the colons. We found an increased macrophage influx into Nur77^-/-^ colons, which is likely a response to the increased MCP-1 levels seen in the colon of these mice. The observed Nur77-mediated repression of MCP-1 mRNA in both Caco-2 and RAW264.7 cells corresponds with the elevated levels seen in Nur77^-/-^ colon. It has been shown in RAW264.7 macrophages that Nur77 potentiates LPS-induced expression of MARCKS, NIK, IKKi, and cyclin D2 mRNA and based on those data it was concluded that Nur77 is a pro-inflammatory nuclear receptor, which does not correspond with our data [50]. In the latter study it was also demonstrated that the expression of these specific genes in peritoneal macrophages derived from WT and Nur77^-/-^ mice was not different after LPS stimulation, indicating that these genes may not be optimal to monitor Nur77-mediated macrophage phenotype changes. In addition, we [29] and others [24,25,30] have shown that Nur77 as an anti-inflammatory function in primary mouse macrophages and RAW cells by repression of NFκB activity.

T-cells were found to be markedly increased in the mucosal area of Nur77^-/-^ mice, which cannot be related to changed expression levels of chemokines, because the specific chemokines regulating migration of certain T-cell subsets to the colonic mucosa remain poorly understood [68]. In the healthy colon, pro-inflammatory T-helper cell subtypes (Th1/Th17) are homeostatically restrained by Foxp3^+^ regulatory T-cells (Tregs) [69]. In IBD Foxp3^+^ Treg cell numbers increase in the intestinal mucosa, reflecting a need to counteract the pro-inflammatory Th1-pressure [70–72]. The TNBS mouse model we used is also Th1-cell driven [38]. It was demonstrated by Neurath et al. [73] that TNBS-colitis resulted in a Th1-mediated transmural infiltrative disease limited to the colon. In addition, it was shown with the TNBS colitis mouse model that disease may be prevented when blocking either IL-12 or CD40Ligand-CD40 interaction [73,74]. Nur77 is known to regulate the Th1/Treg balance and we observed a decrease in Foxp3 expression in TNBS-treated Nur77^-/-^ compared to WT mice [23,75]. In addition, T-cell specific deletion of NR4A family member Nurr1 has been shown to exacerbate DSS colitis by induction of Th1 cells and diminishing Foxp3 expression on Tregs [76]. These latter results suggest that Nur77 is involved in maintenance of the Th1/Treg balance in the gut during experimental colitis.

Azathioprine and its active metabolite 6-mercaptopurine (6-MP) are drugs that are widely prescribed as an effective maintenance therapy in patients with CD [77]. The activity of Nur77 is enhanced by 6-MP, but with limited specificity, because not all immunosuppressive effects of 6-MP involve Nur77 activation [78]. In monocytes/macrophages 6-MP reduces the inflammatory response and capacity of these cells independent of Nur77 and in T-cells, endothelial cells and epithelial cells 6-MP mediates its anti-inflammatory function through Rac1 inhibition [79,80].

In summary, Nur77-deficiency results in aggravated colitis with more neutrophil, macrophage and T-cell infiltration in the gut. To understand the function of Nur77 in the gut, we revealed that Nur77-overexpression results in supression of the inflammatory status of both macrophages and gut epithelial cells. Therefore, we propose a protective role for the nuclear receptor Nur77 in colitis and thus identified a novel target for intervention for which specific agonists need to be identified.

## Supporting Information

S1 ARRIVE Checklist ARRIVE Guidelines Checklist. (PDF)

S1 Fig Flow cytometry gating strategy for macrophage populations in healthy colon. Within the live CD45^+^ colonic lamina propria cells, a CD11b^+^ Ly6G^-^ population was selected and from this gate the CD64^+^ population (macrophages) was further divided based on Ly6C and MHC class II markers. Three different macrophage populations can be discriminated (P1–3).
(EPS)

S2 Fig Inflammatory cytokine levels in mouse colon and plasma. SDF-1α levels were determined in the supernatants of overnight colon cultures from DSS-treated mice (A). Protein levels of a number of cytokines were determined in plasma from DSS treated- (B) and TNBS treated mice (C). Data are presented as mean±SD.
(EPS)

S1 Table Primer sequences used for semi-quantitative real-time PCR. (DOC)

S1 Text Supplemental Materials and methods. (DOC)
