# Supplementary material for: Deficiency of Nuclear Receptor Nur77 Aggravates Mouse Experimental Colitis by Increased NFκB Activity in Macrophages
Source: PLoS One. 2015 Aug 4;10(8):e0133598. doi: 10.1371/journal.pone.0133598 (PMC4524678; doi:10.1371/journal.pone.0133598)
Supplement: S1 Table — (DOC) [file pone.0133598.s004.doc]

**S1 Table.** Primer sequences used for semi-quantitative real-time PCR

| Gene | Forward primer sequence | Reverse primer sequence |
| --- | --- | --- |
| 36B4 | 5’-GGACCCGAGAAGACCTCCTT-3’ | 5’-GCACATCACTCAGAATTTCAATGG-3’ |
| IL-10 | 5’-GCTCTTACTGACTGGCATGAG-3’ | 5’-CGCAGCTCTAGGAGCATGTG-3’ |
| TNFα | 5’-AGATAGCAAATCGGCTGACG-3’ | 5’-AGCGCATGGATCTCAAAGAC-3’ |
| IL-6 | 5’-GTTCTCTGGGAAATCGTGGA-3’ | 5’-GGAAATTGGGGTAGGAAGGA-3’ |
| KC | 5’-CTGGGATTCACCTCAAGAACATC-3’ | 5’-CAGGGTCAAGGCAAGCCTC-3’ |
| MCP-1 | 5’-AGCACCAGCCAACTCTCACT-3’ | 5’-CGTTAACTGCATCTGGCTGA-3’ |
| Foxp3 | 5’-CCCATCCCCAGGAGTCTTG-3’ | 5’-ACCATGACTAGGGGCACTGTA-3’ |
| MIF-1 | 5’-GCCAAGGGTTGACTTCAAGA-3’ | 5’-TTCAGGGTCAAGGCAAACTT-3’ |
| P0^a^ | 5’-TCGACAATGGCAGCATCTAC-3’ | 5’-ATCCGTCTCCACAGACAAGG-3’ |
| MCP-1^a^ | 5’-CCTAGCTTTCCCCAGACACC-3 | 5’-CCCAGGGGTAGAACTGTGG-3’ |
| IL-8^a^ | 5’-TGTTCCACTGTGCCTTGGTTTCTCC-3’ | 5’-TGCTTCCACATGTCCTCACAACATCAC-3’ |
| IL-6^a^ | 5’-CGCCTTCGGTCCAGTTG-3’ | 5’-TCGTTCTGAAGAGGTGAGTG-3’ |
| TNFα^a^ | 5’AGGACACCATGAGCACTGAAAG-3’ | 5’-AGGAGAGGCTGAGGAACAAG-3’ |
| MIP-1α^a^ | 5’-ACGGGCAGCAGACAGTGG-3’ | 5’-GGCGTGTCAGCAGCAAGTG-3’ |
| Nur77^a^ | 5’-GTTCTCTGGACGTCATCCGCAAG-3’ | 5’-GCAGGGACCTTGAGAAGGGCCA-3’ |
| CXCL1^a^ | 5’-TGTTCCACTGTGCCTTGGTTTCTCC-3’ | 5’-TGCTTCCACATGTCCTCACAACATCAC-3' |
| Occludin^a^ | 5’-ACAAGCGGTTTTATCCAGAGTC-3’ | 5’-GTCATCCACAGGCGAAGTTAAT-3’ |
| Claudin-2^a^ | 5’-CGGGACTTCTACTCACCACTG-3’ | 5’-GGATGATTCCAGCTATCAGGGA-3’ |

^a^ indicates human primers
