# Supplementary material for: Deficiency of Nuclear Receptor Nur77 Aggravates Mouse Experimental Colitis by Increased NFκB Activity in Macrophages
Source: PLoS One. 2015 Aug 4;10(8):e0133598. doi: 10.1371/journal.pone.0133598 (PMC4524678; doi:10.1371/journal.pone.0133598)
Supplement: S1 Text — (DOC) [file pone.0133598.s005.doc]

**Supplemental Materials and methods**

**Study design and experimental procedures**

Before starting the experimental procedures sample size neccesary in order to detect a 20% difference in primary outcome was calculated. We needed 9 animals to have sufficient power to detect a 20% difference with a power of 80% and a p-value of 0.05. Primary outcomes of the colitis models were the disease activity index (DAI) and H&E inflammation/histology scores. Secondary, the cellular composition of the inflammation was assessed by immunohistochemistry.

During our experiments mice were weighed every morning to monitor their colitis development and welfare. In addition, all animals in the experiments were evaluated by a welfare diary to see whether any deviations from normal behaviour occur such as: fur, posture, activity and skin. There were no animal dropouts. At the end of the experiment, mice were sacrificed by an intraperitoneal ketamine/xylazine injection depending on the weight of the mice (ketamine = 238mg/kg, xylazine = 24mg/kg).

**Housing**

The mice were housed in individually ventilated cages (IVC; DSS colitis) or standard cages (TNBS) at the specific pathogen free (SPF) animal facility of the Amsterdam Medican Centre. At this facility the light dark cycle (12:12) was strictly regulated and the ambient temperature was kept between 20 and 25^o^C. Animals were kept in social groups from 2 to 6 individuals per cage and were identified by ear marks. The bedding material consisted of autoclaved hardwood material and tissue paper was used as nesting material. Also, a nesting box was provided. Mice had ad libitum access to food (normal chow) and water.
